# What does the future look like for kelp when facing multiple stressors?

**DOI:** 10.1002/ece3.10203

**Published:** 2023-06-26

**Authors:** Brigitte Wear, Nessa E. O'Connor, Matthias J. Schmid, Michelle C. Jackson

**Affiliations:** ^1^ Department of Biology University of Oxford Oxford UK; ^2^ Somerville College Oxford UK; ^3^ School of Natural Sciences, Discipline of Zoology Trinity College Dublin Dublin Ireland; ^4^ School of Natural Science University of Galway Galway Ireland

**Keywords:** conservation biology, global change, marine ecology, stressor interactions

## Abstract

As primary producers and ecosystem engineers, kelp (generally Order Laminariales) are ecologically important, and their decline could have far‐reaching consequences. Kelp are valuable in forming habitats for fish and invertebrates and are crucial for adaptation to climate change by creating coastal defenses and in providing key functions, such as carbon sequestration and food provision. Kelp are threatened by multiple stressors, such as climate change, over‐harvesting of predators, and pollution. In this opinion paper, we discuss how these stressors may interact to affect kelp, and how this varies under different contexts. We argue that more research that bridges kelp conservation and multiple stressor theory is needed and outline key questions that should be addressed as a priority. For instance, it is important to understand how previous exposure (either to earlier generations or life stages) determines responses to emerging stressors, and how responses in kelp scale up to alter food webs and ecosystem functioning. By increasing the temporal and biological complexity of kelp research in this way, we will improve our understanding allowing better predictions. This research is essential for the effective conservation and potential restoration of kelp in our rapidly changing world.

## Graphical abstract

Kelp are globally in decline due to human activity. Here, we give an overview of the role of multiple stressors, such as warming and pollution, and their interactions in driving these declines. We argue that further research is needed with greater biological and temporal realism to improve our understanding of the effects of multiple stressors on kelp for their effective conservation.

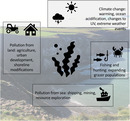

## 1 WHAT ARE KELP?

**FIGURE 1:**
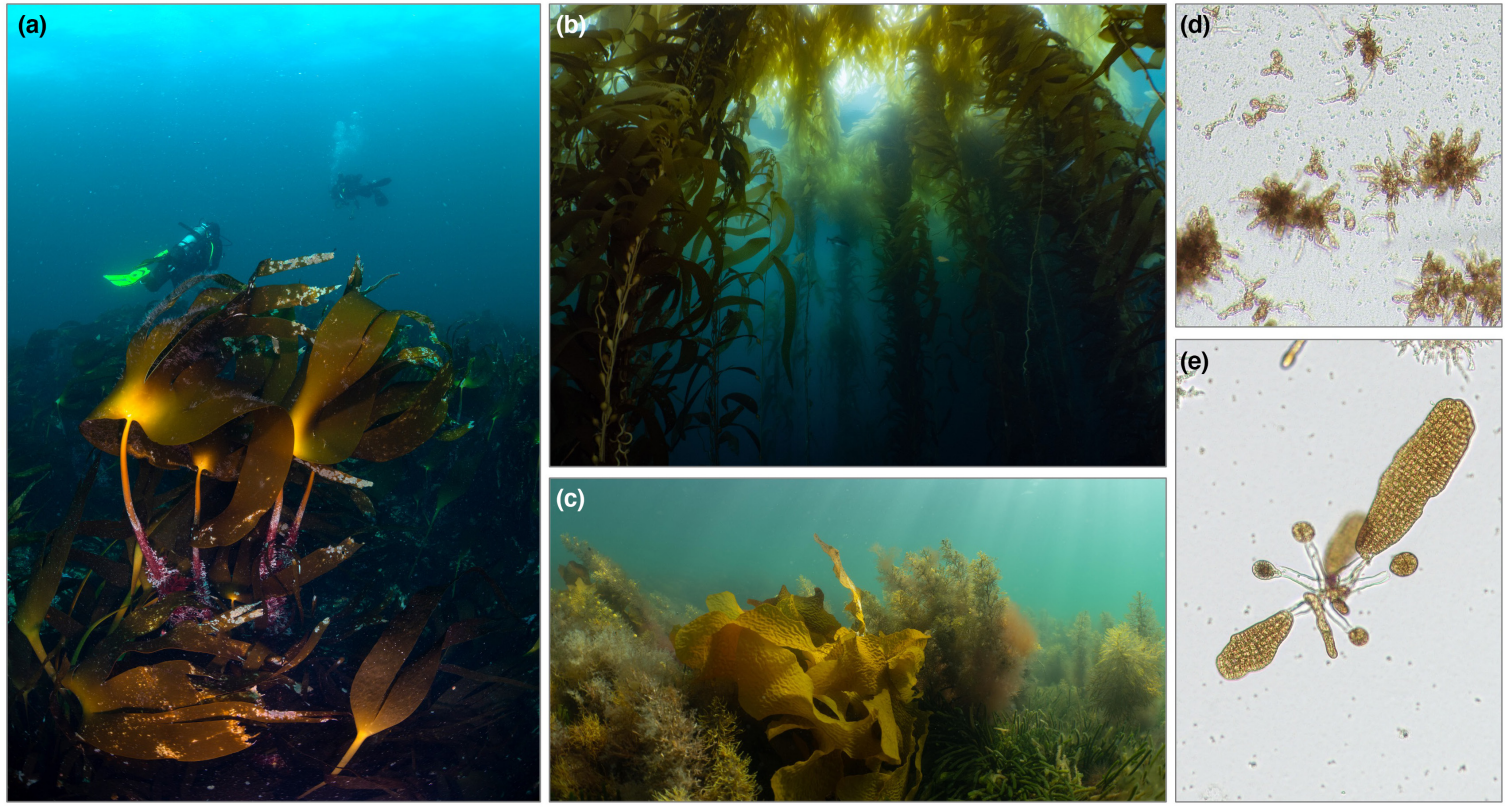
Examples of different kelp species at various life stages and locations; (a) *Laminaria hyperboea* in Ireland, (b) *Macrocystis pyrifera* forest in Tasmania, Australia, (c) *Ecklonia radiata* in Tasmania, Australia. Male and female gametophytes (d) and young sporophytes (e) of *Macrocystis pyrifera*. Pictures by Kenan Chan (a), Joanna Smart (b, c), and MS (d, e).

Kelp is a non‐taxonomic term that refers to ecologically important canopy‐forming large brown macroalgae, usually of the Order Laminariales (although other functionally similar macroalgae are sometimes included; Fraser, 2012), that inhabit hard substrata of the seafloor (Figure 1). Kelp play a key role in primary and secondary productivity through photosynthesis and the export of dissolved and particulate organic material in coastal ecosystems (Paine et al., 2021; Takao et al., 2015), where a large proportion of kelp production enters coastal food webs mainly as detritus (Krause‐Jensen et al., 2018; Krumhansl & Scheibling, 2011; Queirós et al., 2019; Steneck et al., 2002). Kelp form habitats for invertebrates and juvenile fish and also act as ecosystem engineers by altering water flow and sedimentation rates (Smale et al., 2013). The loss of kelp can drive disruptions in ecosystems (Filbee‐Dexter & Wernberg, 2018; Scherner et al., 2012), affecting the abundance and diversity of species, including economically important organisms, such as abalone (Kiyomoto et al., 2013). The kelp life cycle consists of two main life stages, the macroscopic diploid sporophyte and the microscopic haploid gametophyte (Figure 1). The sporophyte produces zoospores, which grow into gametophytes. Gametophytes produce gametes (through gametogenesis), which fuse to form zygotes, maturing into sporophytes (Hurd et al., 2014). Zoospores depend on lipid reserves as an energy source, while gametophytes and subsequent stages are supported by photosynthesis, thus zoospores use different biochemical mechanisms and are often sensitive to different levels and types of environmental stressors compared to other life stages (Leal et al., 2018).

## 2 MULTIPLE STRESSORS IN KELP ECOSYSTEMS

As the human footprint on our planet continues to grow, most ecosystems are subject to multiple simultaneous stressors (Côté et al., 2016). Kelp are affected by stressors, such as rising sea temperature and marine heat waves, invasions by species introductions and range expansions, direct and indirect effects of over‐fishing, algal blooms, ocean acidification, loss of sea ice, and the resulting increase in ultraviolet exposure (Diehl et al., 2020; Donham et al., 2022; Filbee‐Dexter et al., 2019; Hollarsmith et al., 2020; Kroeker et al., 2017; Miranda et al., 2019; Shukla & Edwards, 2017; Starko et al., 2022). Kelp generally live in coastal habitats which are also exposed to stressors from land use including nutrient enrichment, toxins, and sedimentation (Wernberg et al., 2019). For example, near urban coasts, kelp are affected by toxins, such as copper from industrial waste and mine drainage, which decrease the rate of zoospore germination in *Undaria pinnatifida*, *Macrocystis pyrifera*, and *Lessonia nigrescens* (Contreras et al., 2007; Leal et al., 2018). Climate change is having negative effects by, for instance, causing reduced sea ice in polar regions, resulting in increased turbidity, and reduced light penetration. While in some cases shrinking ice caps may initially increase the substrata available for kelp settlement, it has been predicted that kelp growth, productivity, and vertical distribution will ultimately decline in these regions (Bonsell & Dunton, 2018; Filbee‐Dexter et al., 2019). Melting sea ice also locally reduces salinity, and *Alaria esculenta*, *Saccharina latissima*, and *Laminaria solidungula* have strong bleaching and high mortality in low salinities. In contrast, sporophytes of *Laminaria digitata* tolerate a range of salinities and may not be negatively affected (Karsten, 2007). Evidence also suggests that high levels of ultraviolet radiation can reduce growth by damaging biochemical processes in photosynthesis and denaturing DNA (Heinrich et al., 2015; Müller et al., 2008; Xiao et al., 2015).

**FIGURE 2:**
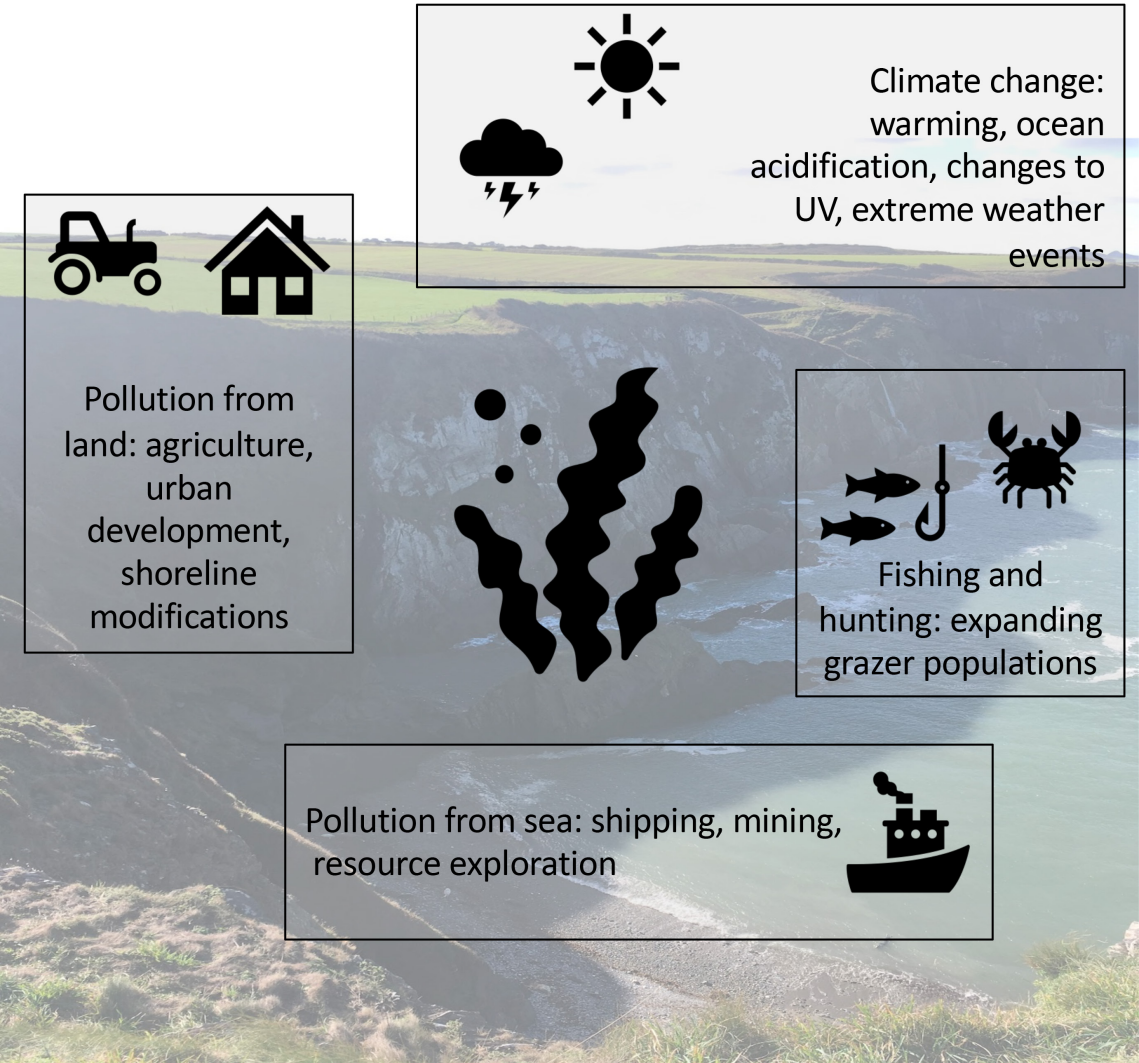
Schematic demonstrating the range of anthropogenic stressors that can affect kelp‐dominated systems. Local stressors include pollution (chemical and nutrient) from the land and sea and expanding grazer populations caused by human exploitation. Global stressors associated with climate change include acidification, changes to ultraviolet radiation, and temperature.

These stressors, which operate from local to global scales, can interact to determine their combined cumulative effect (Figure 2; Falkenberg et al., 2012). Growing evidence suggests that the combined effect of two stressors is rarely additive (i.e., additive = the sum of their parts; Orr et al., 2020, 2022). Instead, stressor pairs frequently result in impacts which are more or less than the sum of their parts (i.e., non‐additive responses). These are known as synergistic and antagonistic stressor interactions, respectively (Jackson et al., 2021; Orr et al., 2020). Evidence suggests that climate warming can exacerbate the negative effects of acidification and pollutants. For instance, warming and acidification combine to inhibit gametophyte growth in *Ecklonia stolonifera* (Gao et al., 2019) and increase mortality of *Macrocystis pyrifera* zoospores (Gaitán‐Espitia et al., 2014), while warming and copper pollution interact synergistically to decrease the size of *Macrocystis pyrifera* gametophytes (but do not affect *Undaria pinnatifida* gametophytes; Leal et al., 2018). Warming also exacerbates damage caused by elevated ultraviolet radiation on sporophyte formation causing reduced egg release in *Saccharina latissima* and *Laminaria digitata* (Müller et al., 2008) and decreases in the zoospore germination rate in *Alaria marginata* (Fredersdorf et al., 2009). In contrast, increased temperature can mitigate the effects of some stressors. For example, warming reduced the negative effects of increased ultraviolet radiation in *Alaria esculenta* by promoting photosynthesis (Roleda, 2009). In comparison, other studies have found that warming exacerbates damage of elevated ultraviolet radiation on sporophyte formation, reduces egg release in *Saccharina latissima* and *Laminaria digitata* (Müller et al., 2008), and decreases the zoospore germination rate in *Alaria marginata* (Fredersdorf et al., 2009). Despite the growing understanding of the effects of multiple stressors on kelp (see Table S1) there are still large areas where further research is needed (Hoffman et al., 2003; Hollarsmith et al., 2020) to understand the growing list of context dependencies (i.e., variation in responses between species, locations, and life stages). Here, we outline knowledge gaps and key questions to help us understand how multiple stressors will affect kelp and shape the future global distribution of this important foundation species.

## 3 HOW DOES THE INTENSITY (OR SEVERITY) OF DIFFERENT STRESSORS AFFECT THE RESPONSE OF KELP POPULATIONS TO MULTIPLE STRESSORS?

Many field and experimental studies are limited in the resolution of the stressor gradient they use, which can cause problems when predicting impacts of stressors that vary in intensity over space and/or time (Vye et al., 2015). For instance, global sea temperatures have increased by 0.1°C per decade since the 1950s (IPCC, 2022), with rates of warming at least two times higher in the Arctic (IPCC, 2022). There is also some uncertainty in the level of warming that will be experienced over coming decades, with predictions ranging (on average) from 1.5 to 7°C (IPCC, 2022). Although some kelp, such as *Saccharina latissima*, have high thermal plasticity (Müller et al., 2008) and may have increased growth rate with warming, other species have reduced growth and productivity with increasing temperature (Table S1). This usually depends on the location, with populations at a latitude with temperatures near their thermal maxima experiencing reduced growth with increasing temperature, while the same species in a location with historical temperatures at the lower end of their thermal limits might experience higher growth rates with warming. However, warmer trailing edge kelp populations in the Northeast Atlantic have been shown to be more thermotolerant than cooler range center populations (King et al., 2018, 2019). The level of warming will also be important when determining the outcome of interactions with other stressors, and vice versa. For instance, with low nutrient concentrations, increasing temperature mitigates the reduced growth rates in juvenile *S. japonica* sporophytes (Gao et al., 2017) and juvenile *Eisenia bicyclis* sporophytes (Endo et al., 2017) but exacerbates the damage in juvenile *Undaria pinnatifida* sporophytes (Gao et al., 2013). In contrast, high nutrient concentrations ameliorate the negative impacts of warming in *Macrocystis pyrifera* (Fernández et al., 2020; Schmid et al., 2020). More research is needed over gradients of multiple stressor severity to make robust predictions about kelp responses to an uncertain future.

## 4 HOW DO MULTIPLE STRESSOR EFFECTS VARY OVER TIME IN KELP POPULATIONS?

Since kelp have distinct life stages, it is important to consider how combined stressor effects on one life stage influences the other stage (Gauci et al., 2022). When considering climate warming, the different life stages of kelp vary in tolerance to increased temperature, for example, *Ecklonia cava* sporophytes can tolerate a larger range than gametophytes (Takao et al., 2015). Similarly, ocean acidification can have contrasting effects on different life stages because of the different mechanisms used to maximize photosynthetic rates: both passive diffusion of CO_2_ as well as the carbon‐concentrating mechanism (Maberly et al., 1992). The use of both mechanisms could explain the lack of response to ocean acidification observed in sporophytes of many kelp species (Fernández et al., 2015; Gordillo et al., 2016; Hollarsmith et al., 2020; Iñiguez et al., 2016; Kang & Chung, 2018; Nunes et al., 2016). However, decreased survival and growth have been observed in *Macrocystis pyrifera* gametophytes under low pH (van der Loos et al., 2019). Zoospores do not rely on photosynthesis for growth; therefore, no effect of decreased pH on zoospore germination in *Macrocystis pyrifera* and *Undaria pinnatifida* has been detected (Leal et al., 2018; Roleda et al., 2012). Gauci et al. (2022) found that gametophyte exposure to warming reduced recruitment and thermal tolerance of juvenile sporophytes in *Laminaria digitata*. However, there is still an open question of how responses to multiple stressors at one life stage are transferred to the next. Here, amplified impacts may be caused by lagged responses to past events or accumulation of stress severity over time (Jackson et al., 2021), as seen in coral reefs (Hughes et al., 2019).

The timing of stressor events can also be important across generations, with historical exposure to stressors altering how organisms respond to current and future stress (Fernández et al., 2021; Jackson et al., 2021; Schmid et al. 2021). For instance, adaptation following stressor events can result in reduced impacts of similar future events on kelp populations (Filbee‐Dexter et al., 2020; Jackson et al., 2021). However, Filbee‐Dexter et al. (2020) found that kelps from degraded and healthy reefs were equally vulnerable to heatwaves. This suggests no effect of previous exposure, but much more research is needed to understand how different stressors interact over time, especially when species interactions are considered (Jackson et al., 2021).

## 5 HOW DO MULTIPLE STRESSORS SCALE UP TO ALTER KELP INTERACTIONS WITH OTHER SPECIES (AND ENTIRE KELP‐BASED FOOD WEBS)?

Although some kelp populations may survive and even increase productivity and resilience with anthropogenic stressors, many populations will decline in diversity and biomass, causing shifts to irreversible alternative states (Christie et al., 2019; Filbee‐Dexter & Wernberg, 2018; Kumagai et al., 2018; Miranda et al., 2019; Veenhof et al., 2022). In nutrient‐enriched and polluted waters, the green algae *Ulva* spp. may increase in abundance at the expense of canopy‐forming kelp (Martins et al., 2014; Russell et al., 2009; Scherner et al., 2012). Nutrient enrichment can also cause phytoplankton blooms, which decreases light penetration to kelp (Filbee‐Dexter et al., 2019), causing high mortality and decreasing growth rate, for example, in *Undaria pinnatifida* juvenile sporophytes (Gao et al., 2013). Alternatively, increased nutrient concentrations might increase kelp growth and resilience against competitive turf‐forming algae (Tamburello et al., 2019), but most of the evidence suggests a negative outcome for kelp. There is also evidence to suggest that over‐fishing of large predators can compound the effects of ocean warming via mechanisms such as range‐shifting grazers and marine heatwaves (Rogers‐Bennett & Catton, 2019; Vergés et al., 2016). However, we still know very little about how multiple stressors interact to alter complex food webs—a question that should be addressed (Kroeker et al., 2017).

Another important stressor in marine ecosystems is the loss of top predators (such as crabs from over‐harvesting or sea stars due to disease), which may lead to an increase in the abundance of herbivores, such as sea urchins, and subsequent over‐grazing of kelp (Gorra et al., 2022; Ling et al., 2009). For example, the hunting of otters in the 19th century reduced otter populations and led to an increase in sea urchin populations, and a subsequent decrease in kelp forest cover. The kelp population recovered after a hunting ban in 1911 with population densities maintained for decades until the decline of otter populations again in the 1990s due to increased predation by whales (Filbee‐Dexter & Scheibling, 2014; Paine, 1980). In Northern California, the dramatic decline in keystone predator population densities (such as the sunflower star, *Pycnopodia helianthoides* due to disease), also led to an increase in sea urchin population densities. Here, marine heat waves interacted synergistically with the loss of the sunflower star, resulting in a decline of the kelp *Nereocystis luetkeana* (McPherson et al., 2021). Ultimately, this can result in a shift from kelp forests to essentially barren grounds (Filbee‐Dexter & Scheibling, 2014; Smale et al., 2013), with implications for the entire food web. However, most studies on the effects of multiple stressors on kelp have focused on individuals at a single life stage and have been principally conducted in the laboratory, and thus may not represent the complexities of communities and ecosystems.

## 6 HOW DO THE IMPACTS OF MULTIPLE STRESSORS AFFECT THE FUNCTIONING OF KELP AND THE ECOSYSTEM SERVICES ASSOCIATED WITH THEM?

Kelp provide many important services for humans. Some kelp species are used for human consumption, play a role in mitigating climate change through carbon sequestration (Dolliver & O'Connor, 2022; Filbee‐Dexter & Wernberg, 2020; Gilson et al., 2021), or assist in climate adaptation by damping waves and protecting coasts (Smale et al., 2013; UNEP, 2023). Recent studies show that the economic value of kelp is at least three times higher than previously estimated (e.g., Northeast Atlantic kelp is valued at $71 k per hectare per year), which is driven primarily by fisheries production and nitrogen uptake (Eger et al., 2023). In a potential negative feedback loop, more regular enhanced wave action from climate change induced cyclones and storms has been shown to cause population losses (Perkol‐Finkel & Airoldi, 2010), but some kelp communities can recover quickly through frequent recruitment and high growth rates (Krumhansl et al., 2016). Other evidence suggests storms may promote kelp growth and increase kelp density, by reducing the diffusion boundary layer and increasing nutrient uptake, dislodging competing epiphytes, and increasing recruitment (e.g., in *L. hyperborean*; Pedersen et al., 2012).

Overall, knowledge on the interactive effect of multiple stressors on the functions and services provided by kelp is limited, with many studies restricted to population‐level responses in unrealistic laboratory trials (Bass et al., 2021; Smale et al., 2013). We need more experiments in complex, semi‐natural settings to fully understand how kelp responses scale up to alter ecosystem functions and services.

## 7 HOW WILL MULTIPLE STRESSORS IMPACT MITIGATION APPROACHES?

Despite variability in the responses of kelp to anthropogenic stressors, global kelp populations have already begun to decline since the 1980s, especially in the South Australian Gulfs, Tasmania, the Gulf of Maine, and the Arctic Sea (Johnson et al., 2011; Krumhansl et al., 2016; Perkol‐Finkel & Airoldi, 2010). Other kelp populations, however, have increased in the past, which may also be attributed to improved local management, such as reduction in local pollution and recovery of sea urchin predators on the west coast of Vancouver Island and Southern California Bight (Krumhansl et al., 2016). The larger variation in local trends compared with the global trend suggests that there is spatial variation in adaptations and resilience of kelp species to anthropogenic stressors (Smale et al., 2013). It also provides hope that management and restoration of kelp ecosystems could be successful with the control of only local stressors. For example, the experimental removal of sea urchins and limpets in eastern Australia led to an increase in the population size of *E. radiata* and *Sargassum sinclairii* after 3 months, compared to sites without the removal of these herbivores (Fletcher, 1987). Controlling or removing local stressors usually leads to such successful restoration outcomes when multiple stressor interactions are synergistic (Brown et al., 2013). Here, the removal of one stressor would result in improvements greater than expected additively. However, some evidence suggests the control of one stressor alone may not be sufficient for kelp restoration (Wilman, 2021). Where two stressors have a negative effect on kelp and interact antagonistically, substantial recovery of kelp may only be observed after the removal of both stressors. Indeed, the removal of only one stressor may even reduce ecosystem fitness further if the presence of the removed stressor mitigated the effects of other stressors (Brown et al., 2013). Therefore, the survival of kelp ecosystems may not be severely affected if a second stressor mitigates the damage from the first, but much more research is needed here as more nature recovery strategies are implemented (Eger et al., 2022).

## 8 CONCLUSION: WHAT DOES THE FUTURE LOOK LIKE FOR KELP?

As sea temperature rises, some kelp begin to approach the edge of their range and experience range contractions (Smale et al., 2013; Vye et al., 2015; Vergés et al., 2016), while temperate‐adapted algae expand into polar regions and outcompete kelp which are less well‐adapted to higher temperatures (Filbee‐Dexter et al., 2019). For example, the distribution of suitable habitats for *Ecklonia cava* is predicted to decrease to between 15% and 45% of current distributions by 2100, and it could become extinct with increased herbivore grazing (Takao et al., 2015). In contrast, some species of Arctic kelp have high optimal temperatures, higher than those predicted to occur with climate change (Filbee‐Dexter et al., 2019), suggesting that an increase in sea temperature may be beneficial to the growth and productivity of some stages in some kelp species. This was shown in Spitsbergen, where the increasing temperature between 1996 and 2014 increased total biomass and stability of the ecosystem (Paar et al., 2019). In parts of Alaska, the species composition of kelp communities has also remained relatively static between 1978 and 2012 despite increasing temperature (Filbee‐Dexter et al., 2019). Although the loss of Arctic sea ice will decrease salinity and increase turbidity locally, Arctic rocky shores may present a large potential for the future niches of kelp as they expand northwards (Filbee‐Dexter et al., 2019; Paar et al., 2019).

The context dependencies in kelp responses to multiple stressors could be further understood using advances in multiple stressor theory, with key areas outlined above. Overall, an increase in complexity (biological and temporal) is needed, alongside a focus on mitigation strategies. Here, the multifaceted concept of stressor similarity could be used to predict the outcome of restoration plans (Orr et al., 2022). There is hope that increasing awareness will remove some of the local stressors on marine ecosystems, such as by reducing coastal pollution and over‐harvesting of predators (Steneck et al., 2002). However, evidence suggests that climate tipping points are dangerously close (Lenton et al., 2019) and that global stressors such as increasing temperature, loss of Arctic sea ice, and ocean acidification may already be irreversible. Population declines will be inevitable, but some kelp species will adapt to anthropogenic stressors, increasing growth and reproduction. However, this may be constrained if some life stages are inhibited. As the effects of stressors on all life stages are still largely unknown for most kelp species, future research must focus on understanding these impacts for effective kelp conservation.

## AUTHOR CONTRIBUTIONS

**Brigitte Wear:** Conceptualization (lead); data curation (equal); formal analysis (equal); investigation (equal); methodology (equal); validation (equal); visualization (equal); writing – original draft (lead). **Nessa E. O'Connor:** Investigation (equal); validation (equal); writing – review and editing (equal). **Matthias J. Schmid:** Investigation (equal); validation (equal); visualization (equal); writing – review and editing (equal). **Michelle C. Jackson:** Data curation (equal); investigation (equal); methodology (equal); project administration (equal); supervision (lead); validation (equal); visualization (equal); writing – review and editing (lead).

## ACKNOWLEDGEMENTS

This work was supported by the Department of Biology at the University of Oxford and Science Foundation Ireland's President of Ireland Future Research Leaders Programme: [18/FR/6198] “Beyond biofuel: Advanced seaweed cultivation for biodiscovery and climate change mitigation”. MS was also funded through a European Commission Marie Sklodowska‐Curie Actions Postdoctoral Fellowship (Project 101066815 ‐ ASPIRE).

## Supporting information


Table S1

## Data Availability

No data are associated with this paper.
