# Supplementary material for: What does the future look like for kelp when facing multiple stressors?
Source: Ecol Evol. 2023 Jun 26;13(6):e10203. doi: 10.1002/ece3.10203 (PMC10293785; doi:10.1002/ece3.10203)
Supplement: Supplementary file 1 — Table S1 [file ECE3-13-e10203-s001.docx]

**Supporting Information for: What does the future look like for kelp when facing multiple stressors?**

Wear et al, Ecology and Evolution

**
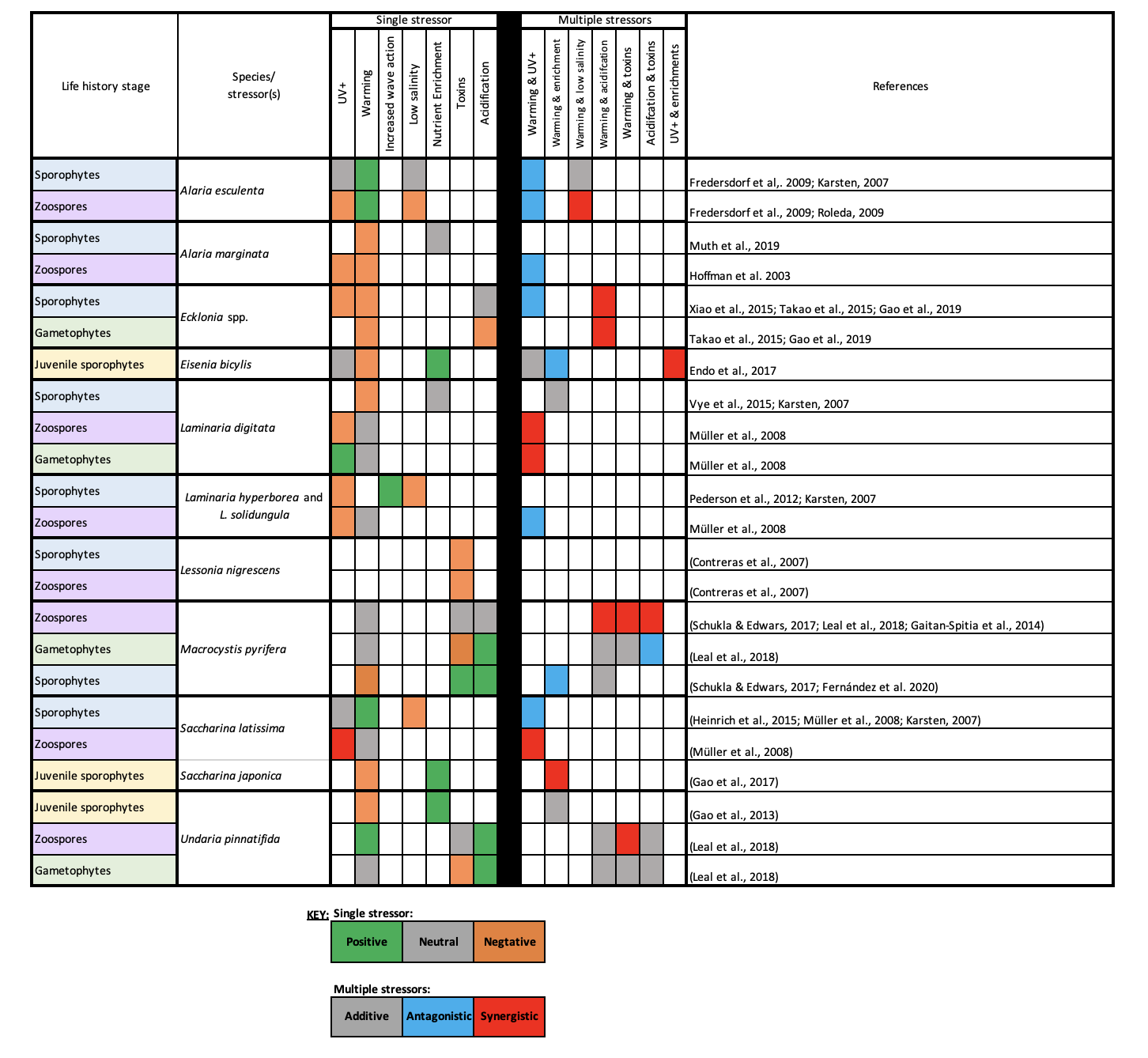
Table S1:** Summary of the effects of anthropogenic stressors and their interactions on adult and juvenile sporophytes, gametophytes, and zoospores of different kelp species. Single stressor effects are shown as negative (e.g., decline in growth or survival with the stressor), positive (e.g., increase in growth or survival with the stressor), or neutral (no change). Interactions are categorised as additive (close to equal to the sum of each stressor when applied alone), antagonistic (less than equal the sum of each stressor when applied alone), or synergistic (more than equal the sum of each stressor when applied alone). The direction (e.g., a positive or negative effect on growth) is not reflected in these definitions. Interpretation of interaction type is either given as categorised by authors of the original paper or interpreted by us based on the definitions above. This is not an exhaustive review of the literature, but a general overview of papers that consider multiple stressors effects for the purpose of starting a discussion about how multiple stressor theory can aid in kelp conservation research.
